# Supplementary material for: Tead1a Initiates Transcriptional Priming Through the TEAD1a/YAP‐Notch1‐Spi1/Cebpα Axis to Promote Neutrophil Fate
Source: Adv Sci (Weinh). 2025 Aug 28;12(41):e05441. doi: 10.1002/advs.202505441 (PMC12591127; doi:10.1002/advs.202505441)
Supplement: Supplementary file 4 — Supplementary Dataset [file ADVS-12-e05441-s004.zip › 3-5.zip!/3-5/02-5.cluster/cluster14/Enrich/ego_MF.pdf]

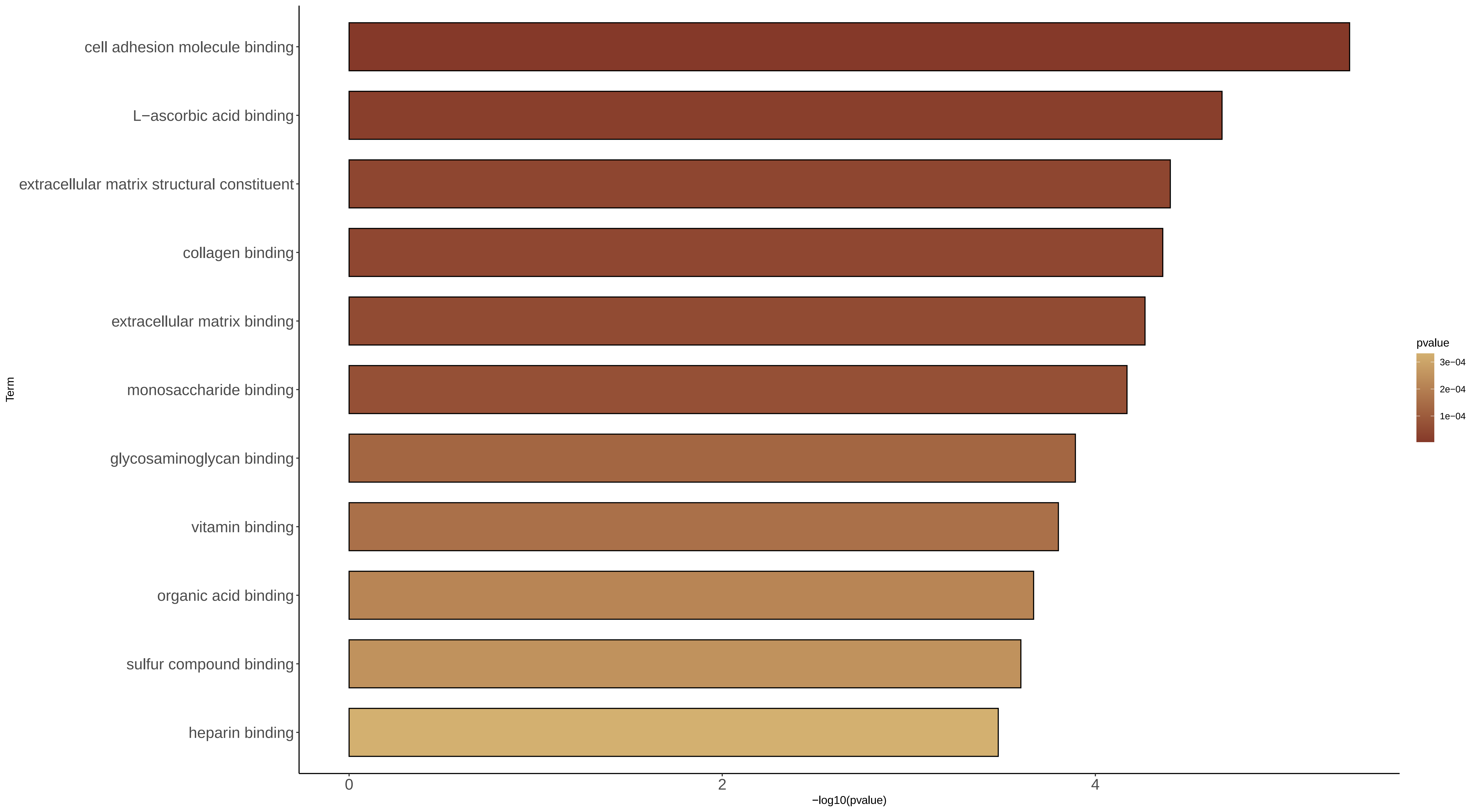
cell adhesion molecule binding
L−ascorbic acid binding
extracellular matrix structural constituent
collagen binding
extracellular matrix binding
monosaccharide binding
glycosaminoglycan binding
vitamin binding
organic acid binding
sulfur compound binding
heparin binding
Term
0
2
4
−log10(pvalue)
pvalue
3e−04
2e−04
1e−04
